# Network localization of regional intrinsic neural activity alterations in migraine and their neurochemical correlates

**DOI:** 10.3389/fneur.2026.1796739

**Published:** 2026-09-03

**Authors:** Hu-Cheng Yang, Si-Jia Bian, Xi-Lei Gao, Feng-Mei Zhang, Ping-Lei Pan, Wen-Hui Li, Zhen-Yu Dai, Si-Yu Gu

**Affiliations:** 1 Department of Radiology, The Yancheng School of Clinical Medicine of Nanjing Medical University, Yancheng Third People’s Hospital, Yancheng, China; 2 Binhai Maternal and Child Health Hospital, Yancheng, China; 3 Department of Neurology, The Yancheng School of Clinical Medicine of Nanjing Medical University, Yancheng Third People’s Hospital, Yancheng, China; 4 Department of Central Laboratory, The Yancheng School of Clinical Medicine of Nanjing Medical University, Yancheng Third People’s Hospital, Yancheng, China; 5 Yancheng Maternal and Child Health Care Hospital Affiliated to Yangzhou University, Yancheng, China

**Keywords:** functional connectivity network mapping, migraine, network localization, resting-state fMRI, visual network

## Abstract

**Background:**

Migraine is a prevalent and disabling neurological disorder. Resting-state functional magnetic resonance imaging (rs-fMRI) studies have investigated regional intrinsic neural activity alterations in migraine but have often yielded inconsistent findings. Emerging network perspectives suggest that brain disorders may be better understood through disruptions in large-scale networks.

**Methods:**

This systematic review included 31 rs-fMRI studies (1,238 migraine patients; 1,005 controls; 302 altered ALFF/ReHo coordinates) to clarify these discrepancies. Using functional connectivity network mapping (FCNM) with Human Connectome Project data (n = 1,093), we identified that heterogeneous regional intrinsic neural activity alterations in migraine converge onto common brain functional networks. Spatial relationships between the identified brain networks and the distribution of major neurotransmitter receptors/transporters were subsequently characterized using the Juspace toolbox.

**Results:**

FCNM analysis revealed that heterogeneous regional intrinsic neural activity alterations reported in migraine studies mapped to common brain functional networks, particularly within visual, somatomotor, and attention systems. The FCNM-identified migraine network exhibited significant spatial correlations with normative distributions of metabotropic glutamate receptor 5 (mGluR5), 5-hydroxytryptamine receptor 2A (5-HT2A), and noradrenaline transporter (NAT).

**Conclusion:**

These findings suggest that intrinsic neural dysfunctions in migraine map to large-scale networks with multi-neurochemical susceptibility. This framework suggests that heterogeneous regional intrinsic activity alterations in migraine are preferentially connected to visual, somatomotor, and attentional systems and are spatially aligned with normative neurochemical maps. These findings are hypothesis-generating and should not be interpreted as evidence of causality or patient-specific receptor alterations.

## Introduction

Migraine is a highly prevalent neurological disorder, affecting over one billion individuals globally and ranking as the second leading cause of disability worldwide, particularly impacting those aged 15 to 49 years (1). Clinically, it is often characterized by debilitating headaches accompanied by symptoms such as nausea, vomiting, and heightened sensitivity to light and sound (photophobia and phonophobia) (2, 3). While the estimated global prevalence is approximately 14–15% (4), significant regional variations exist, with rates reported ranging from 9% in China to as high as 25–35% in Southeast Asia (5, 6). The substantial burden imposed by migraine, reflected in high rates of years lived with disability (7, 8), underscores the critical need for a deeper understanding of its underlying pathophysiology to guide the development of more effective diagnostic tools and therapeutic interventions.

Neuroimaging techniques, particularly resting-state functional magnetic resonance imaging (rs-fMRI) analyses like the amplitude of low-frequency fluctuation (ALFF) and regional homogeneity (ReHo), have been widely employed to explore brain regional functional alterations in migraine (9). These metrics quantify intrinsic neural activity within localized brain regions, providing insights into baseline brain function (10). Metrics for assessing regional spontaneous brain activity include: ReHo, which quantifies the similarity of the time series of a given voxel to those of its nearest neighbors using the Kendall’s coefficient concordance (11), ALFF and its modified version mean ALFF (mALFF), measuring the mean power of low-frequency blood oxygen level-dependent signals (12, 13), fALFF, calculated as the ratio of low-frequency to total amplitude to enhance specificity and reduce noise (12), and dynamic ALFF (dALFF), which captures temporal variability in ALFF typically evaluated through sliding window analysis (14). Numerous studies employing these methods have identified regional abnormalities in functional activity in migraine, often implicating regions crucial for sensory, emotional, and cognitive processes in the pathophysiology of migraine (15–19). However, previous studies have yielded inconsistent results. These inconsistencies may stem from methodological variations in neuroimaging techniques, clinical heterogeneity within patient populations, and the dynamic nature of brain activity across different phases of the migraine cycle (20, 21). Furthermore, these disparities highlight the potential limitations of focusing solely on isolated regional alterations, especially given the emerging consensus that migraine pathophysiology likely involves disruptions within distributed brain networks (22, 23).

Network localization offers a promising framework to address these challenges and synthesize seemingly disparate regional findings (24). The core principle is that structural damage or dysfunction in multiple different brain locations causing the same symptom or disease state often maps to, or perturbs, common functionally connected brain networks. Importantly, when applied to coordinate-based neuroimaging findings, this framework identifies network-level convergence or association rather than proving that the mapped network is causally responsible for the disorder. Instead of examining brain regions in isolation, this approach investigates how focal alterations map onto large-scale brain networks, leveraging normative human connectome data (25–28). By determining if spatially diverse regions exhibiting regional functional changes are part of a common interconnected network, network localization techniques, such as functional connectivity network mapping (FCNM) (25, 26, 29–32), provide a more integrated understanding of brain dysfunction at a systems level. This methodology has successfully mapped symptoms and pathologies in various neurological and psychiatric disorders—including suicide, schizophrenia, cervical dystonia, and Alzheimer’s disease—to specific functional networks (29, 33–35). Notably, it has proven effective in reconciling findings from lesion studies, demonstrating how different lesion locations causing similar symptoms (e.g., hallucinations, movement disorders, and complex behaviors) often perturb a single underlying functional network (36, 37). Furthermore, Burke et al. (38) employed the network mapping approach to identify a common brain network, keyed to the left visual cortex V3/V3A, that functionally links anatomically varied regions of gray matter atrophy in migraineurs, thus unifying disparate observations and suggesting a candidate site for targeted therapy. While network mapping techniques have been applied to structural gray matter alterations in migraine (38), the network correlates of regional functional abnormalities identified in rs-fMRI studies remain unexplored.

Therefore, this study employed FCNM (29), a network localization approach, to investigate whether the diverse regional brain functional alterations in migraine converge onto common brain networks by systematically integrating the spatial coordinates of altered regional findings from previous literature with a large-scale normative functional connectome dataset (Human Connectome Project [HCP]) (39). We hypothesized that the spatially heterogeneous regional alterations reported in migraine rs-fMRI studies represent distinct nodes of a common distributed dysfunctional network rather than isolated and unrelated regional abnormalities. Furthermore, given the well-established involvement of various neurotransmitter systems in migraine pathophysiology (40, 41), we aimed to explore the spatial relationship between the identified FCNM migraine network and the normative distribution of key neurotransmitter receptors/transporters to provide potential neurochemical insights into the vulnerability of these networks. Successfully identifying such network-level convergence and its neurochemical correlates would provide a unifying perspective, potentially reconciling the heterogeneity observed in previous rs-fMRI research and offering novel insights into the distributed network dysfunction.

## Methods

### Literature search and selection

Following the Preferred Reporting Items for Systematic Reviews and Meta-Analyses (PRISMA) guidelines, we conducted a comprehensive and systematic search of the PubMed, Embase and Web of Science databases to identify studies reporting regional functional changes (ALFF, ReHo) in migraine, published prior to September 5, 2024. The search included keywords such as “migraine” AND “ALFF” OR “amplitude of low frequency fluctuation” OR “fALFF” OR “fractional amplitude of low frequency fluctuation” OR “regional homogeneity” OR “dALFF” OR “dynamic amplitude of low frequency fluctuation” OR “ReHo” OR “regional homogeneity.” We also conducted a manual examination of the bibliographies from pertinent review articles and meta-analyses to identify additional relevant studies. A flow diagram of the study selection process is shown in Supplementary Figure S1.

All studies were included according to the following criteria: (a) published in an English-language peer-reviewed journal as an original article; (b) performed a whole-brain comparison between migraine patients and healthy controls; (c) used rs-fMRI analysis reporting ALFF or ReHo results; (d) reported results as coordinates in Talairach or Montreal Neurological Institute (MNI) space. For longitudinal studies, only baseline data were included.

Exclusion criteria were as follows: (a) non-original studies (e.g., review, meta-analysis, meeting abstract); (b) no coordinate system reported; (c) use of regions of interest analysis; (d) involved animal experiments. To avoid data duplication from overlapping patient samples across different publications, only the study reporting the largest sample size and most comprehensive information was retained for analysis. The corresponding author of each included study was contacted via email when additional information was required. Two investigators (Y.H.C. and B.S.J) independently performed literature search and selection and data extraction. Any discrepancies were discussed with another investigator (G.S.Y) until they were resolved.

### Rs-fMRI data acquisition and preprocessing

Our normative functional connectome analysis utilized data from 1,093 healthy adults (594 female; mean [SD] age 28.78 [3.69] years), selected from the HCP 1200 Subjects Data Release (HCP-S1200) (39) following standard quality control.^1^ This cohort consisted of young adults aged 22–37. The HCP’s participant screening excluded individuals based on several criteria, including MRI contraindications, current psychiatric or neurological disorders, psychiatric medication use within the previous 3 months, pregnancy, and a history of head trauma. Specific demographics for the sample analyzed are available Supplementary Table S1. All coordinates extracted from the previous studies were uniformly converted to the MNI standard space. If an original study reported coordinates in Talairach space, established conversion tools^2^ were used to transform them into MNI space.

For this study, we used rs-fMRI data from the HCP (39), acquired on a 3.0 T Siemens Trio system and suitable for in-depth analysis. The acquisition parameters are specified in Supplementary Table S2. Participants were excluded if their imaging data failed quality checks, for example, due to the presence of artifacts or incomplete coverage of the brain.

Rs-fMRI data (see text footnote 1) were preprocessed using SPM12 software^3^ and the Data Processing & Analysis for Brain Imaging (DPABI) toolbox.^4^ The first 10 volumes were excluded from each participant’s scan to permit signal equilibration and allow adaptation to the scanner noise. Slice acquisition timing differences in the remaining volumes were then corrected, followed by a motion correction step via realignment. Head motion parameters for each volume were derived by estimating translational shifts along each direction and angular rotations around each axis. All included participant’s data were within the defined motion thresholds (i.e., maximal translational or rotational motion parameters less than 2 mm or 2°). Additionally, framewise displacement was calculated as an index of inter-volume head motion. To control for nuisance effects, regression analysis was performed using covariates including linear drift, estimated Friston-24 motion parameters, timepoints flagged for excessive motion (framewise displacement > 0.5 mm), and signals derived from global tissue, white matter, and cerebrospinal fluid. Consistent with prior network mapping studies aiming to enhance system-specific correlations, global signal regression was included in the preprocessing pipeline. Then, the datasets underwent bandpass filtering, retaining frequencies between 0.01 and 0.1 Hz. The initial step for spatial normalization involved co-registering each individual’s T1-weighted structural image to their mean functional image. These transformed structural images were then segmented and normalized to MNI space. Subsequently, each filtered functional volume was spatially normalized to MNI space. Finally, all functional data were spatially smoothed using a Gaussian kernel of 6 × 6 × 6 mm^3^ full-width at half maximum.

### FCNM analysis

It should be noted that FCNM differs conceptually from coordinate-based meta-analytic approaches such as activation likelihood estimation (ALE) and seed-based d mapping (SDM). ALE and SDM aim to identify brain regions where reported coordinates spatially converge, thereby localizing consistent regional abnormalities. In contrast, FCNM does not assume spatial convergence of the coordinates themselves; instead, it leverages a normative functional connectome to test whether spatially heterogeneous coordinates are connected to a common network. Accordingly, we selected FCNM rather than ALE/SDM because our primary aim was to test network-level convergence rather than regional spatial convergence.

We utilized the FCNM approach to determine if the reported regional functional alterations in migraine map onto common brain networks. This approach evaluates network-level associations of published peak coordinates using a normative connectome and does not test whether the identified networks are causally involved in migraine. This involved the following steps: First, for each contrast identified in the literature search, spheres with a 4-mm radius were centered at each reported coordinate of significant difference, and these spheres were merged to create a contrast-specific seed mask (henceforth referred to as the ‘contrast seed’). Second, using the preprocessed rs-fMRI data from the 1,093 healthy HCP participants, we computed a functional connectivity (FC) map from each contrast seed to the whole brain for each participant. The procedure entailed calculating Pearson’s r between the average time course of the contrast seed and the time course of all other brain voxels, followed by a Fisher Z-transform to achieve normalization of the resulting correlation values. Third, the participant-level FC maps (*N* = 1,093) were subjected to a voxel-wise one-sample t-test to identify brain regions consistently exhibiting positive FC with each contrast seed across the normative samples. Consistent with previous FCNM studies, we focused solely on positive FC, as the biological interpretation of negative FC remains under debate (42, 43). Fourth, the resulting group-level t-maps were thresholded at *p* < 0.05, corrected for multiple comparisons using a voxel-level false discovery rate (FDR) method, and subsequently binarized. Finally, the binarized connectivity maps corresponding to all included contrasts were overlaid to form a network probability map. This map was then thresholded at 50% overlap (i.e., regions connected to at least 50% of the contrast seeds), a threshold validated in previous FCNM studies (29, 34, 44), to delineate the migraine functional alteration networks.

### Association with canonical brain networks

For ease of interpretability, we examined the spatial relationships between the identified migraine-associated networks and eight well-recognized canonical brain networks. The seven cortical networks included the visual network, somatomotor network (SMN), dorsal attention network (DAN), ventral attention network (VAN), limbic network, frontoparietal network, and default mode network (DMN), as defined by Yeo et al. (45). The Human Brainnetome Atlas (46) was utilized to delineate the subcortical network. The subcortical network mainly includes the amygdala, hippocampus, basal ganglia (including the caudate nucleus, putamen, globus pallidus, nucleus accumbens), and thalamus (46). We quantified these spatial relationships by calculating the proportion of overlapping voxels between each migraine-associated network and each canonical network, relative to the total number of voxels within the respective canonical network.

### Neurotransmitter analysis

To elucidate the relationship between the migraine mapping network and chemoarchitectonic organization, we conducted spatial correlation analysis through 30 PET-derived neurotransmitter transporter and receptor maps available in JuSpace version 1.5^5^ (47). These maps were derived from healthy individuals and therefore represent normative spatial distributions rather than patient-specific receptor or transporter availability. Accordingly, these analyses cannot determine whether any neurotransmitter system is upregulated, downregulated, or functionally altered in migraine. The statistical significance of the correlation was determined using a permutation test of 5,000 iterations across all analyses, corrected by false discovery rate (FDR) with *p* < 0.01.

## Results

### Included studies and sample characteristics

Following the systematic literature search and screening process detailed in Supplementary Figure S1, a total of 31 rs-fMRI studies (302 foci) involving 1,238 patients with migraine and 1,005 healthy controls were included for the subsequent FCNM analysis (9, 14, 18, 19, 48–73). The included rs-fMRI studies utilized ReHo, ALFF, and their variants to assess regional intrinsic neural activity, encompassing ReHo (*n* = 13 studies), ALFF (*n* = 13), fractional ALFF (*n* = 7), mean ALFF (*n* = 1), and dynamic ALFF (*n* = 2). Additionally, the patient samples encompassed a variety of migraine subtypes, including infrequent episodic migraine, frequent episodic migraine, migraine without aura (MWoA), MWoA with anxiety, MWoA during the interictal period, chronic migraine, and vestibular migraine. Detailed sample and imaging characteristics of the included studies are summarized in Table 1.

**Table 1:** Sample and imaging characteristics of the studies included in the study.

Study Indices		Disorders	Subjects (female) Patient/HC		Mean age (SD) Patient/HC		Scanner FWHM threshold Standard space				Foci
Chen et al. (48)	ReHo ReHo ReHo	IEM FEM CM	19 (14) 20 (16) 17 (8)	31 (18) 31 (18) 31 (18)	41.58 (2.56) 38 (2.69) 49.49 (3.55)	49.77 (2.46) 49.77 (2.46) 49.77 (2.46)	3.0 T 3.0 T 3.0 T	8 mm 8 mm 8 mm	FDR (*p* < 0.05) FDR (*p* < 0.05) FDR (*p* < 0.05)	MNI	31
Chen et al. (64)	dALFF	MWoA	21 (16)	21 (8)	31.19 (6.38)	30.19 (6.3)	3.0 T	6 mm	GRF (*p* < 0.05)	MNI	7
Chen et al. (64)	dALFF	MWoA	40 (34)	33 (26)	38.02 (9.79)	33.26 (5.76)	3.0 T	NA	GRF (voxel *p* < 0.001, cluster *p* < 0.05)	MNI	7
Feng et al. (51)	fALFF	MWoA	60 (47)	60 (45)	31.72 (6.65)	29.25 (7.26)	3.0 T	6 mm	FDR (*p* < 0.05)	MNI	8
Dong et al. (65)	mALFF	CM EM	18 (15) 18 (15)	18 (15) 18 (15)	38.83 (11.31) 38.39 (8.88)	38.72 (11.81) 38.72 (11.81)	3.0 T	4 mm	Bonferroni (*p* < 0.05)	MNI	11
Gui et al., (53)	ReHo	EM	27 (25)	30 (26)	15.00,9.00,16.00	16.00,13.50,17.00	3.0 T	6 mm	FWE (*p* < 0.05)	MNI	1
Hodkinson et al. (54)	fALFF	EM	40 (30)	40 (30)	32.7 (9)	NA	3.0 T	6 mm	FWE (*p* < 0.05)	MNI	31
Kim et al. (36)	fALFF	MWoA	44 (44)	31 (31)	36.2 (8.8)	35.2 (9.2)	3.0 T	6 mm	FWE (voxel *p* < 0.001 cluster *p* < 0.05)	MNI	3
Lei and Zhang (55)	ReHo	Migraine-Epi Migraine-Int	19 (14) 22 (17)	22 (16) 22 (16)	35.53 (10.67) 33.32 (10.27)	34.59 (7.99) 34.59 (7.99)	3.0 T 3.0 T	6 mm 6 mm	GRF (*p* < 0.01) GRF (*p* < 0.01)	MNI	2
Lei and Zhang (57)	ALFF	Migraine-Epi Migraine-Int	19 (14) 22 (14)	22 (16) 22 (16)	35.53 (10.67) 33.32 (10.27)	34.59 (7.99) 34.59 (7.99)	3.0 T 3.0 T	6 mm 6 mm	GRF (*p* < 0.01) GRF (*p* < 0.01)	MNI	1
Li et al., (60)	ReHo	MWoA	72 (57)	46 (34)	21.30 (NA)	21.24 (NA)	3.0 T	8 mm	FWE (*p* < 0.05)	MNI	4
Ning et al. (67)	ALFF	MWoA	16 (13)	16 (13)	28.3 (6.0)	27.1 (4.8)	3.0 T	NA	Monte Carlo simulations (*p* < 0.05)	MNI	5
Li et al. (23)	ReHo ALFF fALFF	VM VM VM	17 (10) 17 (10) 17 (10)	17 (10) 17 (10) 17 (10)	NA NA NA	NA NA NA	3.0 T 3.0 T 3.0 T	NA NA NA	FWE (*p* < 0.01) FWE (p < 0.01) FWE (*p* < 0.01)	MNI	3
Liu et al. (59)	ReHo	MWoA	37 (31)	15 (13)	37.97 (9.82)	34.88 (6.66)	3.0 T	6 mm	FWE (*p* < 0.05)	MNI	2
Shi et al. (73)	ReHo	CM	60 (42)	60 (51)	34.7 (7.3)	36.7 (6.5)	3.0 T	6 mm	Alphasim (*p* < 0.05)	MNI	2
Wu et al. (52)	ReHo fALFF	EM EM	24 (17) 24 (17)	23 (17) 23 (17)	31.00,24.00,37.00 31.00,24.00,37.00	27.00,24.00,37.00 27.00,24.00,37.00	3.0 T 3.0 T	4 mm 4 mm	GRF(voxel *p* < 0.001, cluster *p* < 0.05)	MNI	4
Wang et al. (72)	ALFF	Migraine	30 (NA)	24 (NA)	NA	NA	3.0 T	6 mm	Monte Carlo simulations (*p* < 0.05)	MNI	22
Wang et al. (49)	ALFF	VM MWoA	18 (15) 21 (17)	21 (16) 21 (16)	36.17 (8.65) 36.81 (11.61)	36.15 (12.11) 36.15 (12.11)	3.0 T 3.0 T	6 mm 6 mm	FDR (*p* < 0.05) FDR (*p* < 0.05)	MNI	2
Wei et al. (19)	ALFF ReHo	MwoA-A	27 (26)	20 (17)	34.41 (9.75)	33.40 (7.43)	3.0 T	6 mm	Bonferroni (*p* < 0.05)	MNI	2
Wei et al. (50)	ALFF	MwoA	55 (48)	50 (44)	33.58 (10.88)	37.26 (10.28)	3.0 T	6 mm	uncorr	MNI	5
Xiong et al. (9)	ALFF ReHo	VM	57 (50)	88 (72)	46.44 (12.24)	44.69 (13.20)	3.0 T	6 mm	GRF (voxel *p* < 0.005, cluster *p* < 0.05)	MNI	6
Zhang et al. (62)	ReHo	MWoA	22 (13)	22 (13)	41.8 (10.2)	42.0 (10.3)	3.0 T	8 mm	FWE(voxel *p* < 0.001 cluster *p* < 0.05)	MNI	3
Zhang et al. (63)	ALFF	MWoA-DI	27 (19)	29 (13)	34.26 (9.60)	30.90 (10.01)	3.0 T	4 mm	GRF (voxel *p* < 0.001, cluster *p* < 0.05)	MNI	2
Zhao et al. (66)	ReHo ReHo	MWoA-LT MWoA-ST	20 (13) 20 (15)	20 (15) 20 (15)	37.52 (12.2) 27.12 (8.18)	28.4 (8.9) 28.4 (8.9)	3.0 T 3.0 T	4 mm 4 mm	FDR (*p* < 0.05) FDR (*p* < 0.05)	MNI	82
Zhao et al. (68)	ReHo	MWoA	19 (19)	20 (20)	21.8 (2.3)	22.4 (3.1)	3.0 T	NA	FDR (*p* < 0.05)	MNI	22
Li et al. (70)	ALFF	MWoA	62 (48)	42 (34)	21.29 (NA)	21.21 (NA)	3.0 T	6 mm	FEW(voxel *p* < 0.001 cluster *p* < 0.05)	MNI	5
Li et al. (60)	fALFF	MWoA	70 (56)	43 (34)	21.05 (NA)	20.96 (NA)	3.0 T	8 mm	FWE(voxel *p* < 0.001 cluster *p* < 0.05)	MNI	4
Yang et al. (58)	fALFF	MWoA	25 (18)	23 (19)	31.36 (7.47)	32.74 (11.05)	3.0 T	6 mm	FWE (*p* < 0.05)	MNI	3
Yang et al. 58	ALFF	MWoA	50 (50)	50 (50)	28.36 (3.61)	27.60 (3.02)	3.0 T	6 mm	FDR (*p* < 0.001)	MNI	6
Zhe et al. (69)	ALFF	VM	28 (24)	28 (24)	40.18 (10.26)	38.25 (12.47)	3.0 T	6 mm	Bonferroni (p < 0.05)	MNI	2
Zhou et al. (71)	ALFF	MWoA	55 (38)	40 (27)	38.44 (14.0)	41.2 (15.1)	3.0 T	6 mm	FWE(voxel *p* < 0.001 cluster *p* < 0.05)	MNI	14

ALFF, amplitude of low-frequency fluctuations; CM, chronic migraine; dALFF, dynamic amplitude of low-frequency fluctuations; Epi, patients in the episodes phase; fALFF, fractional amplitude of low-frequency fluctuations; FDR, false discovery rate; FEM, frequent episodic migraine; FWE, family-wise error; FWHM, full width at half maximum; GRF, Gaussian random fields; HC, healthy controls; IEM, infrequent episodic migraine; Int, patients in the interictal phase; MWoA, migraine without aura; MwoA-A, migraine without aura with anxiety; MWoA-DI, MNI, Montreal Neurological Institute; Migraine Without Aura during the Interictal period; MWoA-LT, migraine without aura patients with long term; MWoA-ST, migraine without aura patients with short term; NA, not available; ReHo, regional homogeneity; SD, standard deviation; SPM, statistical parametric mapping; uncorr, uncorrected; VM, vestibular migraine.

### Convergent FC associated with regional intrinsic neural activity alterations in migraine

**Figure 1:**
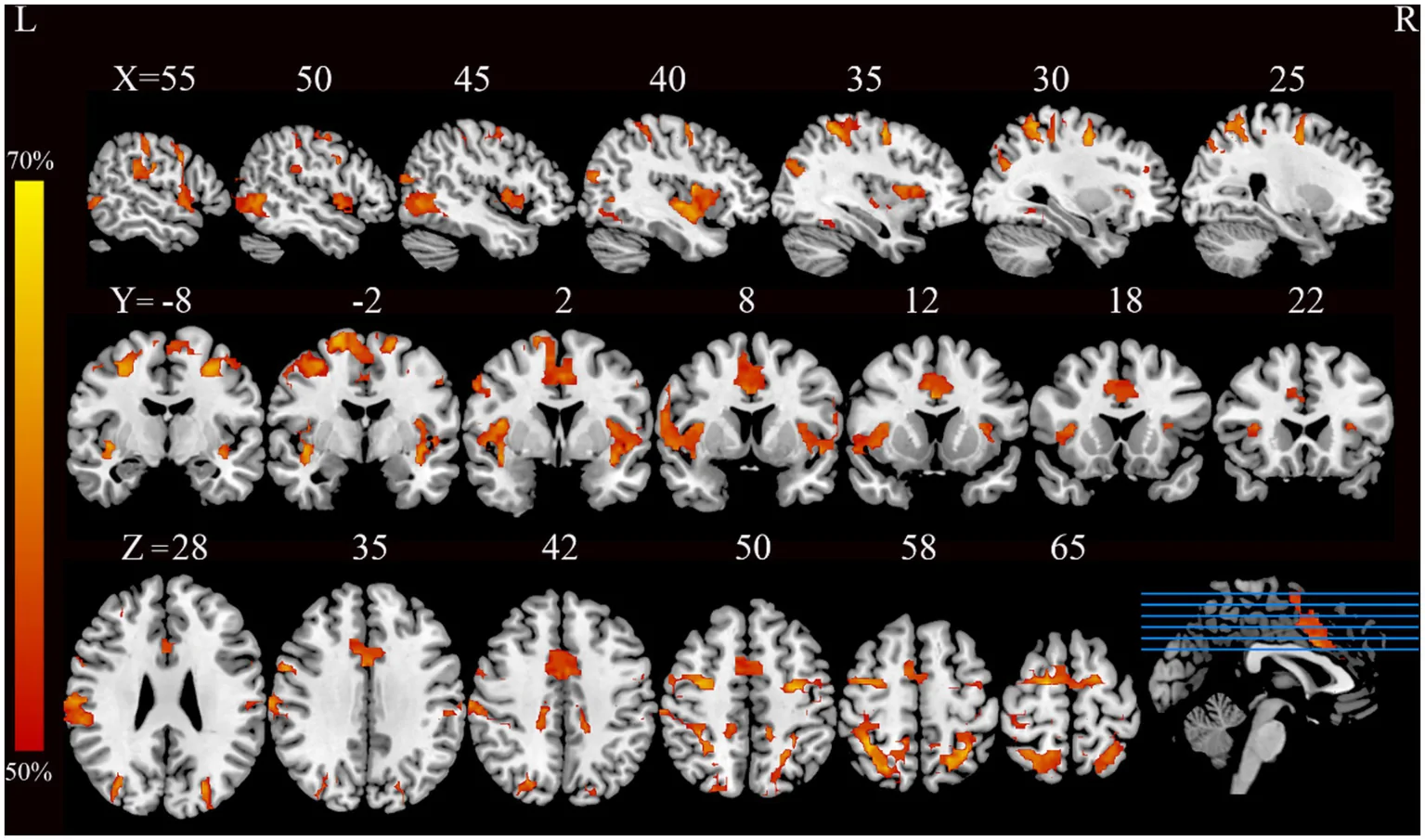
Migraine-associated FC overlap maps based on 4-mm radius sphere. Dysfunctional brain networks are shown as FC probability maps thresholded at 50%, showing brain regions functionally connected to more than 50% of the contrast seeds. FC, functional connectivity; L, left; R, right.

Application of the FCNM approach, integrating coordinates of reported intrinsic neural activity alterations from the 31 rs-fMRI studies with the normative HCP connectome, revealed a convergent pattern of normative FC associated with published regional intrinsic neural activity in migraine. These aberrant FC patterns involved widely distributed brain regions, primarily including the bilateral visual association cortex, bilateral postcentral gyrus, bilateral superior parietal lobule (SPL), and bilateral temporoparietal junction (TPJ) (Figure 1, Table 2).

**Table 2:** Brain FC with altered regional neural activity in migraine.

Brain regions	R/L	Voxels	Brodmann areas	X	Y	Z
Postcentral gyrus	L	155	BA3	-32	−43	59
Postcentral gyrus	R	266	BA3	60	−18	30
Visual association cortex	L	216	BA19/37	−39	−86	21
Visual association cortex	R	169	BA19/37	50	−69	5
Superior parietal lobule (SPL)	L	252	BA7	−24	−56	61
Superior parietal lobule (SPL)	R	281	BA7	23	−56	63
Temporoparietal junction (TPJ)	L	298	BA22/39/40	−57	−31	24
Temporoparietal junction (TPJ)	R	454	BA22/39/40	60	−18	30

BA, Brodmann area; FC, functional connectivity; SPL, superior parietal lobule; TPJ, temporoparietal junction; L, left; R, right.

### Association with canonical brain networks

**Figure 2:**
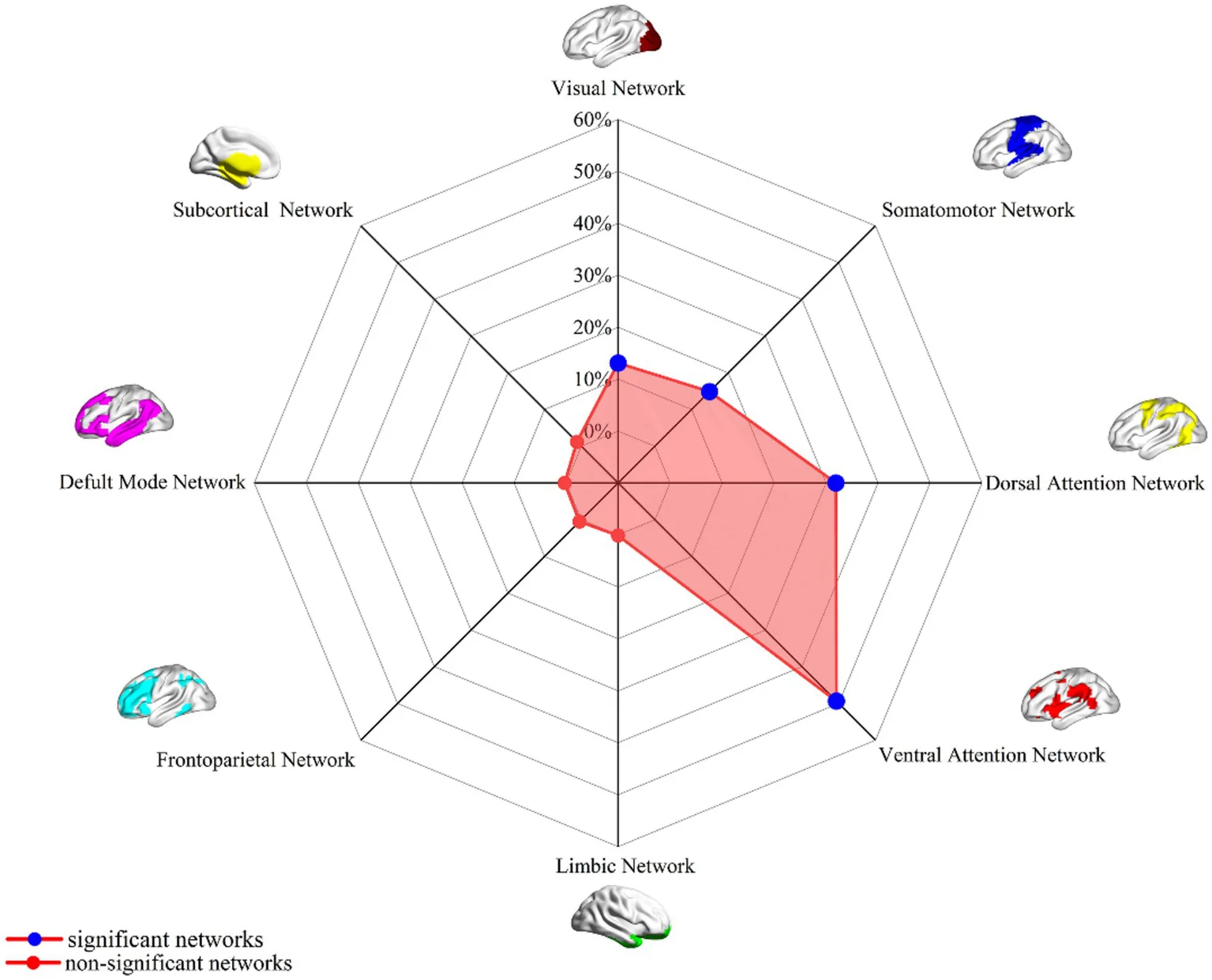
Migraine-associated FC overlap maps based on 4-mm radius sphere in association with canonical brain networks. Polar plots illustrate the proportion of overlapping voxels between each migraine FC map and a canonical network to all voxels within the corresponding canonical network. Note: The blue dot represents brain dysfunction networks, defined as significant networks, exhibiting ≥10% overlap with canonical networks, whereas the red dot represents non-significant networks with <10% overlap. FC, functional connectivity.

Analysis of the spatial overlap between the migraine-related aberrant FC and established canonical brain networks indicated preferential spatial association with specific systems. The network showed the highest overlap with the VAN, largely corresponding to the bilateral TPJ (overlap proportion: 49.4%). Significant overlap was also observed with the DAN, primarily involving the bilateral SPL (31.9%), the SMN, encompassing the bilateral postcentral gyrus (14.9%), and the visual network, including the bilateral visual association cortex (13.2%) (Figure 2). Overlap proportions with the remaining canonical networks (limbic, frontoparietal, DMN, subcortical) were all below 10%.

To further examine whether this network-level distribution differed across intrinsic activity metrics, we performed additional FCNM analyses separately for ReHo and ALFF/fALFF findings. The ReHo-based analysis showed prominent overlap with the SMN, DAN, VAN, and visual network, with overlap proportions of 83.10, 65.21, 42.81, and 40.9%, respectively. In comparison, the ALFF/fALFF-based analysis showed lower but still evident overlap with the DAN, VAN, visual network, and SMN, with overlap proportions of 27.72, 23.92, 10.35, and 4.47%. These findings indicate that the network-level convergence onto visual, somatomotor, and attentional systems is not attributable to a single metric, while also confirming that the two indices differ quantitatively in the strength of their network associations.

### Robustness analyses

FCNM analyses repeated using seed spheres with 1-mm and 7-mm radii yielded topographically similar network patterns to those obtained using the standard 4-mm radius sphere (Supplementary Figures S2, S3, respectively). Furthermore, the pattern of canonical network involvement remained consistent when replicating the FCNM procedure with spheres of 1-mm and 7-mm radii (Supplementary Figures S4, S5, respectively).

### Neurotransmitters associated with neural correlates of migraine

**Figure 3:**
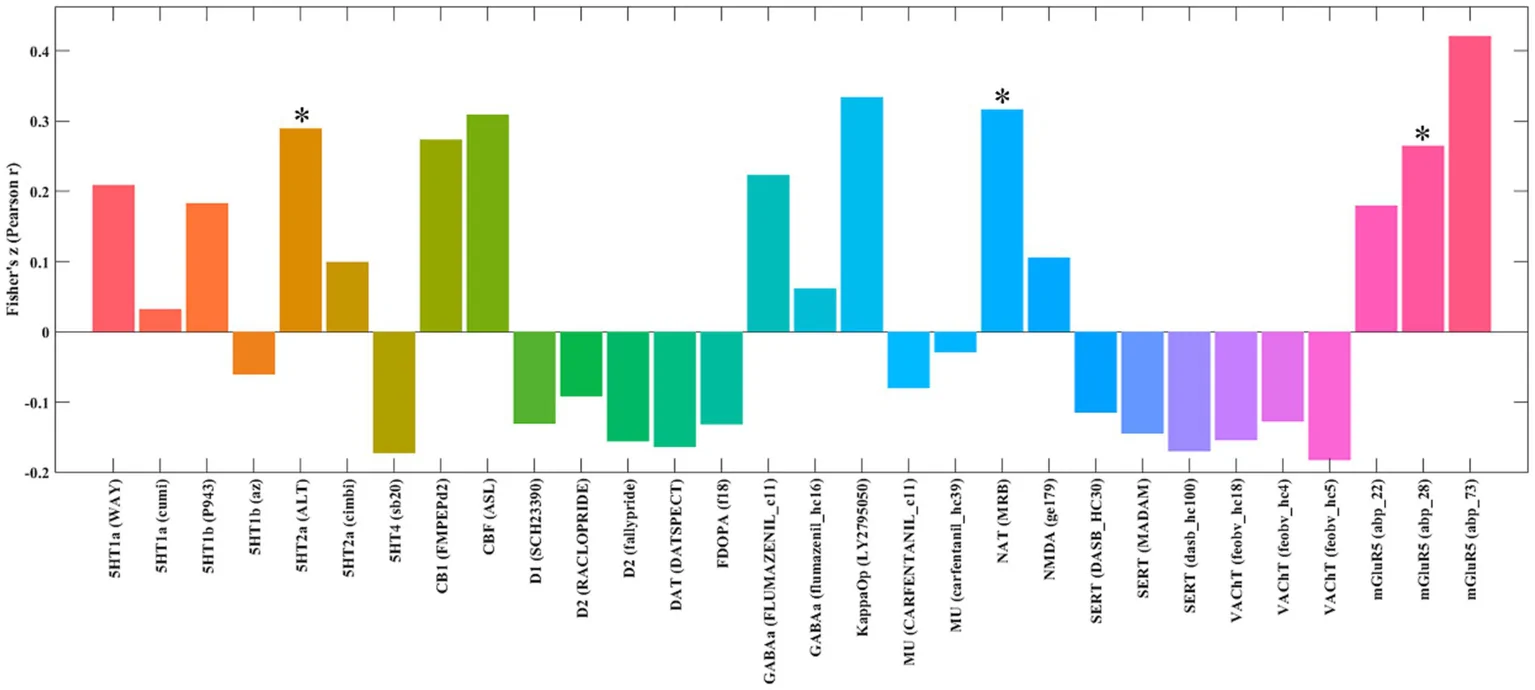
Spatial correlations between the identified migraine-related networks and neurotransmitter distribution maps. *p < 0.01 after false discovery rate (FDR) correction. 5HT1a, 5-Hydroxytryptamine Receptor 1A; 5HT1b, 5-Hydroxytryptamine Receptor 1B; 5HT2a, 5-Hydroxytryptamine Receptor 2A; 5HT4, 5-Hydroxytryptamine Receptor 4; CB1, Cannabinoid Receptor type 1; D1, Dopamine Receptor D1; D2, Dopamine Receptor D2; DAT, Dopamine Transporter; FDOPA, Fluorodopa; GABAa, Gamma-Aminobutyric Acid type A receptor; KappaOp, Kappa Opioid Receptor; MU, Mu Opioid Receptor; NAT, Noradrenaline Transporter; NMDA, N-Methyl-D-Aspartate Receptor; SERT, Serotonin Transporter; VAChT, Vesicular Acetylcholine Transporter; mGluR5, Metabotropic Glutamate Receptor 5.

The FCNM-identified migraine network demonstrated significant positive spatial correlations with the normative distributions of metabotropic glutamate receptor 5 (mGluR5, *p* = 0.0009, *r* = 0.421), 5-hydroxytryptamine receptor 2A (5-HT2A, *p* = 0.001, *r* = 0.289), and the noradrenaline transporter (NAT, *p* = 0.0005, *r* = 0.3168) (Figure 3).

## Discussion

In this study, we integrated the FCNM approach with large-scale resting-state fMRI data from the HCP to map regional functional alterations reported in the migraine literature onto specific common brain networks. Our results demonstrated that the functional networks derived from the FCNM of the regional functional changes primarily involve the nodes within the visual network (notably the bilateral visual association cortex), SMN (bilateral postcentral gyrus), DAN (bilateral SPL), and VAN (bilateral TPJ). The primary network localization was calculated using a 4-mm radius sphere for coordinate seeding, with validation analyses using 1-mm and 7-mm sphere radii yielding robust and consistent results. Our novel exploration of the neurochemical correlates of these networks further revealed significant positive spatial associations with the normative distributions of mGluR5, 5-HT2A, and the NAT. These neurochemical findings should be interpreted as spatial associations with healthy normative maps, not as evidence for patient-specific neurotransmitter abnormalities.

### Visual network dysfunction in migraine

A primary finding of our study was the significant mapping of migraine-associated regional functional alterations onto the visual network. The visual network is critically involved in processing visual information (particularly light and motion) and sensory modulation, and its dysfunction has been strongly linked to migraine pathophysiology and related visual disturbances (74, 75). Research indicates that migraine has a genetic basis, with certain genetic variations potentially influencing visual system development and function, thus increasing migraine risk (76). Consistent with our finding, multiple neuroimaging modalities, including fMRI, structural MRI, and electroencephalography, have revealed alterations within the visual network of migraineurs. These encompass changes in intra-network connectivity and disrupted functional coupling with other brain networks, such as the DMN, executive control network, and AN (50, 77–79). Specifically, prior research indicates structural alterations in the visual cortex of migraine patients, such as changes in cortical thickness and gray matter volume (GMV) (80–82). Furthermore, gray matter atrophy regions associated with migraine exhibit negative FC with visual cortex area V3/V3A, implicating V3/V3A as a critical network hub and suggesting its potential role in migraine pathophysiology (38). Functionally, the visual network may exhibit hyperactivation or enhanced connectivity during the interictal period (83, 84), and differential activation patterns under visual stimulation compared to healthy individuals (77). These functional and structural abnormalities within the visual system are closely associated with characteristic clinical manifestations of migraine, including photophobia, visual aura, and visual snow syndrome (75, 77, 80, 85, 86). Furthermore, disrupted FC patterns linking the visual network with cognitive systems (DMN, executive control, attention, and salience networks) are increasingly recognized as potential underlying mechanisms contributing to migraine pathogenesis (77–79). However, our FCNM results, by showing that disparate regional functional alterations converge onto the visual network, reinforce the centrality of this network dysfunction in migraine. Although the present findings suggest an association between migraine-related functional abnormalities and the visual network, further studies using patient-level imaging data and longitudinal or interventional designs are needed to determine whether this network has diagnostic or therapeutic relevance.

Within the visual network, the visual association cortex integrates visual information with motivational states and learning to guide goal-directed behavior (87, 88). Neuroimaging studies converge on its importance in the pathophysiology of migraine. Structural alterations, notably cortical thickness, have been observed in the visual cortex of migraine patients (80, 81). Functionally, this region exhibits hyperexcitability, particularly evident in persistent visual aura and during negative emotional processing (75, 89, 90). These alterations specifically within the visual cortex are strongly correlated with migraine symptoms like photophobia and visual aura (91), and may also contribute to comorbid psychiatric conditions (92).

### SMN dysfunctional in migraine

Our study also found that regional functional alterations associated with migraine map onto the SMN. The SMN integrates sensory information from the body and environment to guide motor output (93). A growing body of evidence suggests that this network exhibits both functional and structural abnormalities in migraineurs, potentially contributing to migraine pathogenesis (94). Within the network itself, long-range connections between the primary sensory cortex and higher-order association cortices are impaired (95), possibly underlying the sensory hypersensitivity experienced by migraineurs. However, analyses of local FC suggest that migraine involves complex network-level alterations beyond primary sensory cortex dysfunction (96). Furthermore, altered FC between the SMN and other key brain networks, including the DMN, visual network, AN, and salience network, has been observed (21, 97–100). These network alterations appear to vary with different migraine subtypes, disease stages, and clinical features (e.g., pain intensity, attack frequency, comorbid tinnitus), potentially influencing pain perception, cognitive function, emotional regulation, and multisensory integration. Importantly, preliminary studies suggest that therapies like anti-Calcitonin Gene-Related Peptide (anti-CGRP) monoclonal antibodies may modulate SMN FC (101), offering potential mechanistic insights and novel therapeutic targets. Using the FCNM, our study demonstrated that regional functional alterations mapped significantly onto this network, further supporting its involvement in somatosensory processing in migraine. Nevertheless, the coordinate-based nature of the analysis precludes direct inference about the full spatial extent or causal role of SMN dysfunction in migraine.

The postcentral gyrus, a key component of the SMN, serves as the primary somatosensory cortex, processing multimodal sensory information and contributing to motor planning (102). Converging evidence indicates functional and structural alterations in this region in migraine patients, affecting FC, cortical thickness, and GMV (103–105). FC studies indicate enhanced coupling between the postcentral gyrus and pain-related regions, such as the thalamus and brainstem, implicating its role in pain perception, modulation, and the emotional-cognitive dimensions of migraine (103). Structural studies provide evidence of cortical thickness alterations in the postcentral gyrus, correlating with disease duration and headache frequency (104, 105). Additionally, metabolic and iron deposition studies reveal aberrant cerebral blood flow, neural activity, and increased iron accumulation in the postcentral gyrus in chronic migraine (106, 107).

### AN dysfunctional in migraine

Our study also demonstrated significant mapping of regional functional alterations onto both the VAN and DAN. The VAN comprises the temporoparietal junction and ventral frontal cortex, and is typically implicated in stimulus-driven attentional control (108). Accumulating evidence strongly suggests a significant role for the VAN in the pathophysiology of migraine. Within the VAN, regions such as the orbitofrontal cortex, inferior frontal gyrus, right TPJ, and anterior cingulate cortex exhibit aberrant FC and activity patterns, suggesting disrupted intra-network information processing in migraine (95, 109–111). Importantly, aberrant connectivity has also been reported between the VAN and other networks (e.g., executive control, DAN, visual, DMN, salience networks) in migraine (78, 95, 112). These findings support the notion that VAN dysfunction is an integral element of the widespread brain network dysregulation in migraine, potentially impacting attentional allocation and information integration (97, 113). Our finding of the highest network overlap (49.4%) with the VAN underscores its potential significance. The DAN is primarily responsible for top-down, goal-directed attentional control, and spatial orienting (114). Migraine research indicates alterations within this network as well. For instance, migraine without aura has been associated with decreased time-averaged correlations in specific DAN regions (115), while migraine with aura may exhibit increased dynamic fluctuations in DAN connectivity (116). Furthermore, aberrant DAN connectivity is observed during executive attention tasks in migraineurs, suggesting impaired goal-directed attentional function (115, 117). Beyond intra-network changes, migraine also appears to disrupt interactions between the DAN and other networks, such as the reduced FC with the DMN and visual network (78, 79). Subtype-specific alterations are also noted; for example, vestibular migraine is characterized by increased DAN connectivity with the salience network and decreased connectivity with executive control and auditory networks (113, 118). Mechanistically, dysfunction within both the VAN and DAN may be associated with sensory hypersensitivity, pain-biased attention, and cognitive dysfunction in migraine (95, 110, 112). From a therapeutic perspective, interventions such as acupuncture may alleviate migraine symptoms by modulating AN activity (119), suggesting the AN as a potential target for future therapeutic intervention.

Regional alterations reported in the TPJ and SPL contributed substantially to the mapping results for the VAN and DAN, respectively, in our FCNM analysis. The TPJ is primarily involved in higher-level cognitive functions such as attention orienting, self-awareness, theory of mind, and social cognition (120, 121). Studies have shown altered FC of the TPJ in migraine, including increased connectivity with the temporal pole and modulation of connectivity with the locus coeruleus following vagus nerve stimulation (122, 123). Crucially, TPJ dysfunction appears strongly associated with attentional deficits in migraine (124, 125), potentially serving as a therapeutic target (123). The SPL is crucial for spatial awareness, sensorimotor integration, and higher-order cognitive functions (126). A substantial body of evidence suggests that the pathogenesis of migraine is closely associated with functional or structural abnormalities in the SPL (103, 127, 128). Evidence links migraine pathogenesis with functional or structural SPL abnormalities, including potentially decreased FC with the red nucleus and thalamus, possibly are related to pain processing and multisensory integration (103, 129). Structural changes in the SPL have also been linked to headache characteristics (128, 130).

### Neurochemical correlates of the migraine-associated functional networks

Our neurochemical mapping revealed a particularly compelling and statistically robust spatial correlation between the FCNM-defined migraine-associated functional alteration networks and the normative distribution of the mGluR5. Glutamate, the principal excitatory neurotransmitter in the central nervous system, and its diverse receptor subtypes, are increasingly implicated in the core pathophysiology of migraine, from the initiation of attacks and cortical spreading depression (CSD) to the establishment of chronic pain states and central sensitization (131). The mGluR5 receptor, a Group I metabotropic receptor predominantly located postsynaptically and coupled to Gq/11 proteins, plays a significant role in modulating neuronal excitability, synaptic plasticity, and pain processing (40, 132). Its involvement in migraine is supported by a growing body of evidence that mGluR5 is implicated in the modulation of trigeminovascular nociception, the generation and propagation of CSD (133, 134), and significantly, in the mechanisms driving central sensitization, which contributes to symptoms like allodynia and the chronification of migraine (135). The significant spatial alignment we observed, particularly within the visual, SMN, and attentional networks highlighted by our FCNM, therefore strongly suggests that brain regions inherently rich in mGluR5 are preferentially susceptible to the excitotoxic and maladaptive plastic changes that characterize migraine network dysfunction.

Our analysis also identified a significant, albeit slightly less strong, spatial correlation between the migraine-associated functional networks and the normative distribution of the 5-HT2A. The 5-HT2A is recognized as a pivotal modulator in migraine pathophysiology (136–138). Distributed widely in the cerebral cortex, particularly within visual and somatosensory regions implicated in migraine symptoms, as well as in platelets and vasculature, its activation typically leads to neuronal excitation (139, 140). This excitatory role is thought to contribute to cortical hyperexcitability, a potential substrate for CSD and migraine aura (136, 141, 142), with some genetic studies and receptor-informed FC analyses suggesting an association between 5-HT2A and aura or visual network alterations (143–145). Furthermore, 5-HT2A receptors are implicated in pain processing and sensitization (142), and critically, have been shown to be upregulated in chronic headache and medication-overuse headache, potentially via nitric oxide-dependent pathways, thereby contributing to headache persistence and exacerbation (138, 146, 147). Our finding of a significant spatial correlation between migraine-associated functional brain networks and the normative distribution of 5-HT2A receptors lends further support to this, suggesting that brain regions inherently rich in these receptors are preferentially vulnerable to the network dysfunctions seen in migraine, a notion underscored by the antagonist effects of some prophylactic migraine medications at this receptor (140–142).

Expanding this neurochemical profile, our analysis identified a third significant positive spatial correlation between the FCNM migraine networks and the normative distribution of the NAT. NAT is pivotal for noradrenaline reuptake, thereby controlling the intensity and duration of noradrenergic signaling (148). Crucially, common migraine triggers like stress are intricately linked to cerebral metabolism and oxidative stress, with the noradrenergic system serving as a primary mediator of the physiological stress response (41). The observed spatial congruence with NAT distribution therefore suggests that the FCNM-identified migraine-vulnerable networks (including visual, SMN, and attention systems) are under substantial noradrenaline influence, and alterations in noradrenergic tone or clearance, potentially mediated by NAT, may contribute significantly to their functional impairment. This aligns with documented fluctuations in sympathetic activity and noradrenaline levels in migraineurs, particularly in response to stressors (149, 150), and is further supported by the prophylactic efficacy of beta-blockers, which modulate noradrenaline signaling (151, 152). This interpretation is further supported by the prophylactic efficacy of serotonin–noradrenaline reuptake inhibitors (SNRIs) such as venlafaxine and duloxetine, which increase synaptic noradrenaline availability through NAT inhibition and have demonstrated benefit in migraine prevention (151). The convergence of these three distinct neurochemical markers onto the FCNM-identified migraine networks paints a picture of a complex interplay of excitatory and modulatory neurotransmitter systems.

## Limitations

Several limitations should be considered when interpreting the findings of this study. First, the analysis relies on coordinate-based data extracted from published studies, which represents a summary statistic (peak location) and inherently loses spatial information compared to analyses using full statistical maps or individual participant data (24, 44, 153, 154). Consequently, the spatial extent, effect size, and uncertainty associated with the original neuroimaging findings could not be incorporated into the present FCNM framework. Therefore, our findings should be interpreted as network-level convergence patterns associated with reported peak alterations, rather than as a direct or comprehensive localization of migraine-related functional abnormalities. Second, we utilized a normative connectome derived from healthy adults (HCP dataset) to map the networks associated with migraine-related regional functional. While this is standard practice in network mapping (24, 44, 155, 156) and evidence suggests sample selection often has minimal impact on the topography of network localization (25, 155, 157), using a connectome derived from a sample more closely matched to the migraine populations might potentially yield slightly different results, though previous work suggests topographic differences are often minor (154, 157, 158). Third, due to the limited number of studies stratified by migraine subtypes within the existing literature reporting ALFF/ReHo alterations, network localization analyses were not conducted separately for different subtypes. This limits the specificity of the identified network, which represents an aggregate across various migraine forms. Future FCNM analyses with sufficient subtype-specific data are needed to address this. Fourth, the input data were heterogeneous, derived from studies using different ALFF and ReHo calculation methods and acquired on various scanners. While FCNM aims to find convergence despite such heterogeneity, these variations introduce noise and may obscure more subtle network effects. Fifth, methodological choices such as the radius of the coordinate spheres (though sensitivity analyses showed robustness) and the probability threshold (50%) for defining the final network represent specific parameters (24, 38). Exploring alternative parameters could provide further insights but was beyond the scope of this initial network localization. Sixth, the current analysis did not incorporate negative findings from the original studies, nor did it weight studies based on sample size. The potential influence of unaccounted confounding factors (e.g., demographic variations, differences in scanning parameters and analytical process across studies) also cannot be fully discounted. Seventh, the PET-derived neurotransmitter maps were obtained from healthy individuals and therefore represent normative spatial distributions rather than patient-specific receptor/transporter availability. Consequently, the present findings cannot determine whether these neurochemical systems are upregulated, downregulated, or functionally altered in migraine. Eighth, because this study was based on reported peak coordinates, negative studies could not be incorporated, and studies were not weighted by sample size or effect size. These factors may introduce reporting bias and allow studies contributing more foci to disproportionately influence the results. Therefore, our findings should be interpreted cautiously as exploratory network-level convergence patterns rather than definitive evidence of a migraine-specific network. Future studies should validate these findings using leave-one-study-out sensitivity analyses and weighted coordinate-based approaches. Finally, our findings should be viewed within the context of the persistent challenges affecting reproducibility in neuroimaging, including small sample sizes, clinical heterogeneity, experimental variability, and analytical flexibility (159, 160). Continued efforts towards standardization, larger sample sizes, and transparent reporting are crucial for advancing the field.

## Conclusion

In conclusion, by employing the FCNM approach integrating large-scale human brain connectome data from the HCP, this study identified convergent large-scale network associations of intrinsic neural activity alterations in migraine. We demonstrated that heterogeneous regional intrinsic neural activity changes consistently map onto the functional networks such as the visual network, SMN, DAN, and VAN. The involvement of these networks offers a systems-level perspective that may relate to key clinical features frequently reported in migraine, such as visual disturbances, sensory hypersensitivities, altered pain perception, and attentional difficulties, although these links remain associative and require further validation. Our novel exploration of the neurochemical correlates of this FCNM network further reveals significant positive spatial associations with the normative distributions of mGluR5, 5-HT2A, and NAT. Taken together, by demonstrating convergence onto specific large-scale networks and their neurochemical susceptibility, network localization offers a comprehensive framework that may help reconcile previously inconsistent findings regarding regional functional changes in migraine and address concerns about reproducibility. These findings are correlational and coordinate-based, and should be regarded as hypothesis-generating; nonetheless, they may inform future studies aimed at clarifying the network- and system-level organization of migraine pathophysiology.

## Data Availability

The original contributions presented in the study are included in the article/Supplementary material, further inquiries can be directed to the corresponding authors.

## Footnotes

1
https://www.humanconnectome.org/study/hcp-young-adult/data-releases

2
https://www.sdmproject.com/utilities/?show=Coordinates

3
https://www.fil.ion.ucl.ac.uk/spm/

4
https://rfmri.org/DPABI

5
https://github.com/juryxy/JuSpace

## Glosssary

5-HT2A: 5-hydroxytryptamine receptor 2A
ALFF: altered amplitude of low-frequency fluctuation
AN: attention network
anti-CGRP: anti-Calcitonin Gene-Related Peptide
dALFF: dynamic ALFF
DAN: dorsal attention network
DMN: default mode network
DPABI: Data Processing & Analysis for Brain Imaging
DPARSF: Data processing assistant for resting-state
EEG: electroencephalography
FA: flip angle
FC: functional connectivity
FCNM: Functional connectivity network mapping
FEM: frequent episodic migraine
fALFF: fractional ALFF
FOV: field of view
FWHM: full-width at half maximum
GMV: gray matter volume
GRE-EPI: gradient-recalled echo-Planar Imaging
GRF: Gaussian random fields
HC: healthy controls
HCP: Human Connectome Project
mGluR5: metabotropic glutamate receptor 5
MNI: Montreal Neurological Institute
MWoA: migraine without aura
NAT: noradrenaline transporter
PRISMA: Preferred Reporting Items for Systematic Reviews and Meta-Analyses
ReHo: regional homogeneity
ROI: regions of interest
rs-fMRI: resting-state functional magnetic resonance imaging
SD: standard deviation
SMN: somatomotor network
SPL: superior parietal lobule
SPM: Statistical Parametric Mapping
TE: echo time
TR: repetition time
VAN: ventral attention network
