# Supplementary material for: Network localization of regional intrinsic neural activity alterations in migraine and their neurochemical correlates
Source: Front Neurol. 2026 Sep 3;17:1796739. doi: 10.3389/fneur.2026.1796739 (PMC13581937; doi:10.3389/fneur.2026.1796739)
Supplement: Supplementary file 1 [file Supplementary_file_1.docx]

**Network localization of regional intrinsic neural activity alterations in migraine and their neurochemical correlates**

**Supplementary Material**

**Figure S1. Flow chart showing the process of study identification and exclusion**

fMRI, functional magnetic resonance imaging.

**Figure S2.** **Migraine-associated** **FC overlap maps based on 1-mm radius sphere**

Dysfunctional brain networks are shown as FC probability maps thresholded at 50%, showing brain regions functionally connected to more than 50% of the contrast seeds.

FC, functional connectivity; L, left; R, right.

**Figure S3.** **Migraine-associated** **FC overlap maps based on 7-mm radius sphere**

Dysfunctional brain networks are shown as FC probability maps thresholded at 50%, showing brain regions functionally connected to more than 50% of the contrast seeds.

FC, functional connectivity; L, left; R, right.

**Figure S4.** **Migraine-associated** **FC overlap maps based on 1-mm radius sphere in association with canonical brain networks**

Polar plots illustrate the proportion of overlapping voxels between each migraine FC map and a canonical network to all voxels within the corresponding canonical network. Note: The blue dot represents brain dysfunction networks, defined as significant networks, exhibiting ≥10% overlap with canonical networks, whereas the red dot represents non-significant networks with <10% overlap.

FC, functional connectivity.

**Figure S5.** **Migraine-associated FC overlap maps based on 7-mm radius sphere in association with canonical brain networks**

Polar plots illustrate the proportion of overlapping voxels between each migraine FC map and a canonical network to all voxels within the corresponding canonical network. Note: The blue dot represents brain dysfunction networks, defined as significant networks, exhibiting ≥ 10% overlap with canonical networks, whereas the red dot represents non-significant networks with <10% overlap.

FC, functional connectivity.

**Table S1.** Demographic information of the HCP

| **Dataset sample size** | **Age (years)** | **Gender (F/M)** |
| --- | --- | --- |
| HCP 1093 | 28.78±3.69 | 594/499 |

Age is expressed as mean ± standard deviation. Note: HCP, Human Connectome Project; F, female; M, male.

**Table S2.** Resting-state fMRI parameters of the HCP

| **Parameter** | **HCP** |
| --- | --- |
| Scanner | 3.0T Siemens Trio |
| Sequence | GRE-EPI |
| TR (ms) | 720 |
| TE (ms) | 33.1 |
| FA (°) | 52 |
| FOV (mm²) | 208×180 |
| Matrix size | 104×90 |
| Slice thickness (mm) | 2 |
| Slice gap (mm) | 0 |
| Slices | 72 |
| Time points | 1210 |

HCP, Human Connectome Project; GRE-EPI, gradient-recalled echo-Planar Imaging; FA, flip angle; fMRI, functional magnetic resonance imaging; FOV, field of view; TE, echo time; TR, repetition time.
